# Highlights of 2025: advances in germinal centers

**DOI:** 10.1111/imcb.70131

**Published:** 2026-05-14

**Authors:** Louise MC Webb, Michelle A Linterman

**Affiliations:** ^1^ Babraham Institute Cambridge UK; ^2^ Malaghan Institute of Medical Research Wellington New Zealand

**Keywords:** B lymphocytes, germinal center, T cells, T follicular helper cells

## Abstract

The germinal center (GC) is the engine room of the humoral immune response, driving the evolution of B cells into memory and long‐lived plasma cells that can protect against (re)infection. Understanding GC biology remains a key research focus for promoting lifelong health. In this annual update, we highlight several influential studies on GC biology published in 2025 (Fig. 1). Space constraints prevent us from discussing in depth all published work.

## Graphical abstract

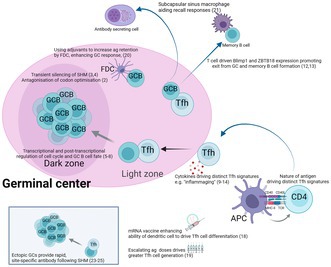

## INTRINSIC MECHANISMS GOVERNING GC B CELL RESPONSES

Within germinal centers, iterative rounds of somatic hypermutation (SHM), positive selection and clonal expansion drive affinity maturation and evolution of the B cell repertoire.^1^ However, targeted DNA damage is risky business. SHM can antagonize codon optimization, thereby reducing the translational stability of mRNA and impacting antibody secretion. Although the biased targeting to complementary determining regions helps minimize this effect, the need for diversity appears to take precedence over codon optimization.^2^ The rapid proliferation of dark zone (DZ) GC B cells comes with increased opportunities for acquisition of detrimental mutations. Ideally, high‐affinity B cells should expand while maintaining a stable genotype. Shortening the G0/G1 phase—the window in which SHM occurs—transiently silences SHM, delaying it until the G0‐like phase that follows their final round of division in the GC dark zone.^3^, ^4^

The light zone (LZ) to DZ transition relies on rapid protein oscillations that cannot be driven by transcription alone. Post‐transcriptional regulation, mediated by microRNAs and RNA‐binding proteins (RBPs), coordinates epigenetic remodeling, metabolism, survival and DNA repair in GC B cells. The RBPs ZFP36L1 and ZFP36L2 are now known to control a cell‐cycle regulon essential for GC expansion; their loss triggers replication stress, DNA damage responses and poor survival—conditions likely to impair SHM.^5^

Our understanding of the epigenetic and transcriptional regulation of GC B cells continues to evolve. The Setd1A/B‐associated factor, Cfp1, was shown to be required for GC entry, proliferation and SHM, sustaining Bcl6, Mef2b and Pou2af1 expression through H3K4me3‐mediated activation of cell‐cycle programs.^6^ Blimp1 was also found to regulate cell‐cycle dynamics and competitive fitness when expressed in a subset of GC B cells.^7^ Additionally, Blimp expression drives the downregulation of Bcl6 and upregulation of IRF4 expression, enabling exit from the GC.^7^ Regulated exit is equally critical for memory formation. The transcription factor, ZBTB18, influences exit from the GC by promoting memory precursor B cell survival.^8^ Like Blimp1, ZBTB18 expression is driven by T cell help. These studies link T cell help to GC contraction and memory B cell formation, highlighting their importance in the termination of the GC response.

## T FOLLICULAR HELPER CELLS

The quantity and quality of Tfh cells are key determinants of GC responses, and their phenotype is shaped by the immune stimulus and their environment. These features are often used to subset Tfh cells, but this may mask their complexity. A broad transcriptional survey of Tfh cells elicited by different types of infections identified a core Tfh signature distinct from T effector cells in both mice and humans. Further specialization identified an early bifurcation into either Tfh or T effector lineages, followed by secondary diversification.^9^ Cytokines are key drivers of Tfh states. For example, TRAF3‐deficient T cells have impaired cytokine sensing resulting in impaired Tfh development^10^; during influenza infection, IL‐6Rα deletion in T cells reduced Tfh numbers and antibody responses^11^; and in LCMV infection, IFN‐γ suppressed Tfh differentiation, reinforcing the early Th1/Tfh bifurcation and suggesting that limiting IFN‐γ may permit coexistence of both lineages.^12^

Tfh plasticity extends to Th17 cells. In Peyer's patches, many Th17 cells can convert to a Tfh state via c‐MAF expression and cues from segmented filamentous bacteria.^13^ Circulating counterparts to these Tfh17 cells are seen in rheumatoid arthritis patients, and their expansion with age suggests roles for these cells in autoimmunity and age‐associated autoreactive antibody production. Age‐associated inflammation (“inflammaging”) further rewires Tfh differentiation, promoting autoreactivity while weakening protective immunity. Aged Tfh cells adopt a hyperactivated, functionally impaired state.^14^ Elevated circulating mitochondrial DNA in older individuals, or in metabolic or autoimmune disease, activates the NLRP3 inflammasome which drives antibody production and glomerulonephritis in mice via increased Tfh cell generation.^15^

The existence of Tfh cells with distinct transcriptional and/or cell surface phenotypes suggests there are opportunities to steer Tfh differentiation to different states that enable optimal GC responses, tailored to the pathogen and/or host and offering more targeted vaccination strategies.

**Figure 1:**
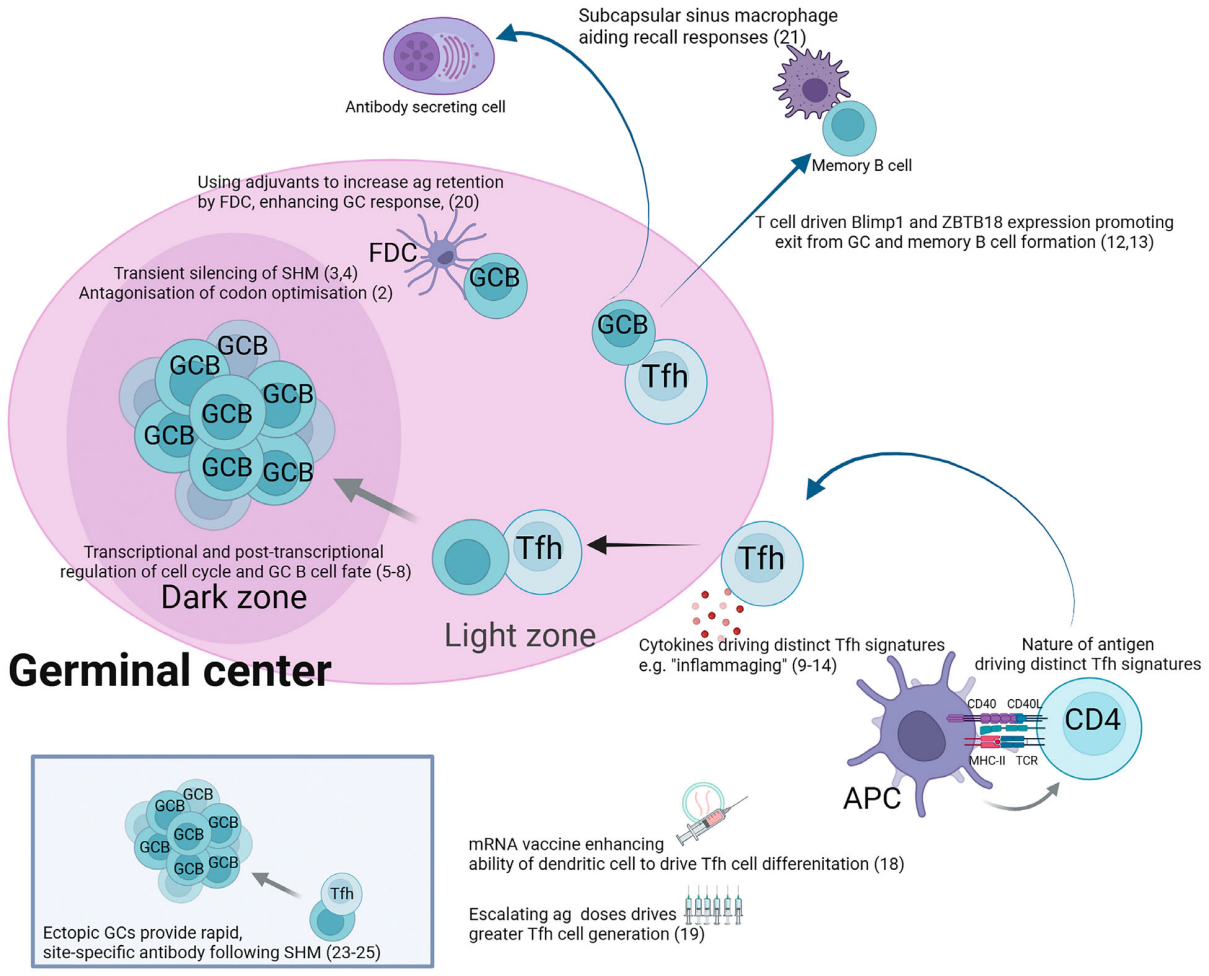
Graphical summary of research advances made in 2025 in germinal center (GC) biology. GCB, germinal center B cells; Tfh, T follicular helper cell; APC, antigen presenting cell; FDC, follicular dendritic cell; SHM somatic hypermutation. Created in BioRender. Webb, L. (2026) https://BioRender.com/bn9jx9v [Correction added on 16 June 2026, after first online publication: Figure 1 has been added, which was previously represented as a graphical TOC image.]

## MANIPULATING THE GC RESPONSE FOR BETTER VACCINATION OUTCOMES

Harnessing our understanding of GC biology to improve vaccine efficacy remains a priority. Novel vaccine platforms include advanced adjuvants; nanoparticle‐based antigen delivery; AI‐guided immunogen design; and next‐generation mRNA or viral‐vector vaccines. Murine models remain a versatile and tractable platform for early‐stage vaccine development. Murine models show that although pre‐plasma cells are heterogeneous in affinity, it is the high‐affinity cells that contribute most to circulating antibody because of greater Tfh‐supported clonal expansion prior to leaving the GC.^16^ Furthermore, low‐affinity B cells can bind the same epitopes as high‐affinity clones, effectively “piggy‐backing” on the T cell help elicited by high‐affinity clones.^17^ These advances suggest that intra‐GC competition is more dynamic and permissive than previously assumed.

The deployment of mRNA vaccine platforms has enabled the rapid synthesis and scalable production of tailored antigen‐encoding constructs, yet we have much to learn about how they work. Lipid nanoparticle encapsulated mRNA vaccines can promote Tfh differentiation through both the adjuvant activity of the lipids and through the mRNA‐driven interferon production, enhancing the ability of dendritic cells (DCs) to initiate Tfh cell differentiation.^18^

Escalating‐dose vaccination strategies are also emerging as a promising approach, especially in the generation of broadly neutralizing antibodies. They elicit a larger and more sustained Tfh response, retaining proliferative capacity and clonal diversity up to 6 months after immunization.^19^ It appears that early antigen exposure can drive greater Tfh expansion before peak antigen delivery, suggesting that vaccine strategies that maximize early Tfh recruitment/expansion can enhance GC output.

Engineering adjuvants to increase antigen persistence is also being leveraged to improve GC output. Phosphoserine‐tagged immunogens combined with alum and saponin nanoparticles enhance lymph flow and antigen entry, resulting in larger GC and antibody responses.^20^ The combined adjuvants enhance intact antigen deposition on FDCs via CR1/2, and saponin nanoparticles deplete CD169^+^ subcapsular sinus macrophages (SSMs), increasing antigen accessible for FDC capture. This may limit recall responses as SSMs are essential for recall immunity, with draining lymph nodes holding memory B cells that mount stronger recall responses and re‐enter GCs more readily upon boosting.^21^

The vaccination site remains a key determinant of the quality of the immune response, particularly for pathogens that enter through mucosal surfaces. IgA is the most abundantly produced antibody isotype at mucosal barrier sites. A sequential class‐switching model has been proposed that links the specificity of mucosal IgA and systemic IgG1 responses to gut‐derived antigens.^22^ Antigen‐specific IgA plasma cells initially arose through a GC‐independent pathway, which predominates for several weeks before being replaced by GC‐derived cells.

## ECTOPIC GCs

GC‐like structures outside secondary lymphoid organs (SLOs) are common in older individuals and arise in chronic inflammation, autoimmunity, persistent infection and cancer. Despite their simpler architecture, recent work confirms that they support key GC functions.

Although they take longer to form than conventional GCs, ectopic GCs enable cognate interactions with rapid, site‐specific, antibody production and can support affinity maturation and export memory B cells directly into tissue.^23^ GC formation has also been observed in nasal turbinates after influenza infection.^24^ Analysis of nasal‐associated lymphoid tissue GCs revealed that GC collapse during fatal infection is associated with inflammatory skewing of Tfh cells toward a Th1‐like state that cannot support GC B cells, highlighting the importance of the environment in maintaining Tfh responses.^25^

## CLOSING REMARKS

These insights into GC biology emphasize how the environment and the immunogen can be used to manipulate GC responses (Fig. 1). Metabolic, epigenetic, transcriptional and post‐transcriptional programs shape GC output through their action on GC‐associated cells and structures. The discoveries of 2025 have direct implications for tuning immunotherapies and vaccine‐induced GC responses to promote lifelong health.

## CONFLICT OF INTEREST STATEMENT

Michelle Linterman reports funding from GSK outside of this work.

## AUTHOR CONTRIBUTIONS

**Louise MC Webb:** Conceptualization; writing – original draft; writing – review and editing. **Michelle A Linterman:** Funding acquisition; writing – review and editing.
